# COVID-19, anorexia nervosa and obese patients with an eating disorder - some considerations for practitioners and researchers

**DOI:** 10.1186/s40337-021-00369-w

**Published:** 2021-01-20

**Authors:** Mladena Simeunovic Ostojic, Joyce Maas, Nynke M. G. Bodde

**Affiliations:** 1 Center for Eating Disorders, Mental Health Center Region Oost-Brabant, Wesselmanlaan 25a, 5707 HA Helmond, The Netherlands; 2 Department of Medical and Clinical Psychology, Tilburg University, Warandelaan 2, P.O. Box 90153, 5000 LE Tilburg, The Netherlands

**Keywords:** COVID-19, Eating disorders, Anorexia nervosa, Obesity

## Abstract

Since COVID-19 is a global health emergency, there is an urgent need to share experiences on decision-making with regard to safety recommendations and for hypotheses that can inform a more focused prevention and treatment. Moreover, combining research into eating disorders and obesity with research into COVID-19 may provide a unique opportunity to shed light on the susceptibility to COVID-19.

To date, there are no case series available that report on associations between pre-existing eating disorders (ED) and COVID-19 due to, among other reasons, the lack of widespread testing, standardized data collection, and a potential sampling bias (i.e., having COVID-19 symptoms and being hospitalized increases the chances of being selected for testing). In the absence of current observational data on the incidence of COVID-19 in ED, a thorough knowledge of infection risk, common and uncommon manifestations of COVID-19, as well as an in-depth understanding of the clinical features and pitfalls in the diagnosis of viral infections in ED is of vital importance. Below, we briefly summarize the current data on the risk of COVID-19 in ED populations and propose recommendations for safety interventions and future research.

## The risk of COVID-19 infection in patients with ED - safety recommendations

Several studies suggest that both obesity and underweight can increase the risk of viral infection in a U-shaped manner, which holds in particular for influenza-related pneumonia [1]. Paradoxically, despite serious malnourishment and alterations in immune functions, it has been reported that anorexia nervosa (AN) seems to be associated with fewer symptomatic viral infections [2–4]. However, because of the paucity, contradictions and methodological shortcomings of previous studies [2–5], extreme care must be taken in drawing any firm conclusion.

As the incidence of asymptomatic viral infections in patients with AN has not been studied, it is unknown whether they are less susceptible to viral infections, or whether these patients have a normal or even increased susceptibility to infection with a more asymptomatic or mild disease course and an absence of conventional clinical markers of infection [3, 6]. Indeed, it has been hypothesized that immune exhaustion in the context of persistent, spontaneously elevated levels of pro-inflammatory cytokines in AN may lead to an impaired capacity to mount an acute-phase response to infection under an extra stimulus ([7], for review). Additionally, one recent study revealed a significant negative genetic correlation between AN and the acute phase infection marker C-reactive protein (CRP) [8]. From a clinical point of view, this is of particular concern as asymptomatic acute viral infection does not preclude direct damage caused by viruses and consequently secondary bacterial infections, necessitating timely diagnosis and antibacterial therapy. Actually, a number of studies do confirm that AN is associated with increased morbidity and mortality due to delayed diagnosis of bacterial infections, occult sepsis and atypical infections [3, 6, 9–11]. Indeed, and commonly seen, a reduced or absent fever and CRP response, and a delayed leukocytosis may contribute to delays in the diagnosis of infection in AN [3, 6]. In addition, AN may have clinical features that can mimic COVID-19, such as gastrointestinal symptoms, syncope, myalgias, and fatigue. Furthermore, laboratory abnormalities such as lymphopenia, thrombocytopenia, abnormal liver function and elevated ferritin, interleukin 6 (IL-6), and creatine kinase levels can be seen in COVID-19 [12, 13], but are also associated with AN [14]. To further add to the complexity, using body temperature for detecting acute infection in AN during refeeding is difficult because of lower basal body temperature, blunted fever response, but increased production of body heat during renutrition [15]. This suggests that the standard temperature threshold ≥37.8 °C for infection no longer holds, and that a temperature elevation of ≥1.1 °C for defining fever [16] must be interpreted in the context of the possible thermic effect of renutrition in AN. Importantly, resting energy expenditure increases by 10% during asymptomatic viral infection [17], and in symptomatic infection fever increases energy requirements by 10–13% for each degree of temperature increase. The hosts’ ability to endure the infection-related (hyper) metabolism may well be affected by their insufficient energy-nutrient intake and low body reserves, and might be additionally hampered by COVID-19’s gastrointestinal symptoms.

A particular concern exists regarding the vulnerability of patients through different stages of recovery. The clinical features of COVID-19 infection vary from asymptomatic to critical illness, with the latter associated with overactive inflammatory immune responses, leading to a cytokine storm and acute respiratory distress syndrome (ARDS) [12]. The association between COVID-19 and intense cytokine release raises the possibility that the “immunosuppressed” patient with acute AN is paradoxically protected or may at least not be predisposed to a poorer outcome. Indeed, most of the literature to date indicates that unlike other viral agents, COVID-19 seems not to be more severe in immunosuppressed patients although contradictory results have emerged depending on various other factors and the underlying cause of immune suppression (including the specific disease and treatment, the time of application, as well as the type and dose of medication) (e.g., [18–20]). Based on previous research (e.g., [20]), it is not unreasonable to believe that patients with acute AN are not immune to COVID-19, and when infected, especially if presenting an asymptomatic and mild disease, as it seems to be likely, they may be underdiagnosed. Conversely, since an aberrant inflammatory profile is recently found to be a state marker associated with acute AN and/or low BMI (i.e., which was not seen in those who had recovered from AN) [21], infection could be reactivated during the recovery phase, in particular during the refeeding process. Hypothetically, it might have occurred due to a suboptimal control of COVID-19 infection during acute AN (silent infection), leading to impaired viral clearance, and allowing a second episode of viral replication and/or a greater immune activity and consequently clinical infection during renutrition. On the one hand, refeeding could be helpful as it increases the number of circulating lymphocytes, which should improve the defence against the viral infection; but on the other hand, it could also be harmful as with refeeding sequestered infected lymphocytes could be released into the bloodstream, and as the circulating viral load increases, the disease reactivates. In line with this, several reports show impaired viral clearance and COVID-19 reactivation in patients with compromised immune systems after withdrawal of some immunosuppressive drugs [22, 23]. Also, severe and atypical infections are among the features of the refeeding phase that are frequently reported in the literature, but are poorly understood and often overlooked [9]. Nevertheless, the association of nutritional status and refeeding with the risk and course of infections has attracted great research attention recently. Noteworthy, hypophosphatemia has been reported as an independent risk factor for the development of infections by impairing high-energy substrate availability for host defense [24, 25] and has been observed to correlate with lymphocyte count and severity of COVID-19 [26]. Additionally, one recent study demonstrated that the increased incidence of medical complications including infections in severe AN during the first 30 days of re-feeding, compared with the 30 days prior to treatment and the second phase of treatment, is accounted for less by the starvation process per se, and more by a transient process of physiological adaptation to refeeding [27]. Furthermore, the first randomized controlled trial on refeeding among adult mechanically ventilated ICU patients found that caloric restriction significantly reduced the incidence of major infections, in particular respiratory infections [25]. Collectively, these findings reinforce the hypothesis that clinically overt and more severe COVID-19 infection could arise, at least in part, as a response to refeeding. Determining the possibility of asymptomatic infection, impaired viral clearance and reactivation of the virus has important implications for coping with COVID-19 in AN. Hence, a combination of cycle threshold values of RT-PCR reactions as direct measures of COVID-19 viral loads on different time courses of infection and immunodiagnostic serology tests is crucial for the interpretation of other accurate diagnostic methods. Because persistent detection of low viral load by RT-PCR may have little clinical implication as both ‘live’ and ‘dead’ viruses are detected, future research may wish to investigate viral culture for detecting clinically significant viral shedding in this patient population.

Conversely, obesity, defined as body mass index (BMI) > 30, appears to be an independent risk factor for more severe COVID-19-related infection and mortality. Importantly, an increase of visceral adipose tissue (VAT) and fat mass percentage – and not only BMI – correlate positively with COVID-19 morbidity [28]. Angiotensin-converting enzyme 2 (ACE2) has been identified as the host cell-surface receptor for COVID-19, forming a basis for viral tropism in several cells including adipocytes. Although obesity does not appear to have an effect on ACE2 expression by adipocytes [29], increased numbers of ACE2-expressing cells, due to higher adipose tissue (AT) volume, may increase susceptibility to viral host-cell entry [28]. ACE2 may also be shed into circulation and may modify pulmonary susceptibility of obese people to SAR-CoV-2 infection [30]. As viral tropism for and utilization of AT as a reservoir has already been shown for many types of viruses (e.g., H5N1, adenovirus AD-36, HIV), it is speculated that obese patients infected with COVID-19 may be potentially more contagious than lean individuals [28]. In support of this hypothesis, one not yet published study [31] shows that the COVID-19 virus can infect human adipocytes as well as that the viral load is three times higher in aged adipose cells.

Together, clinical evidence and research suggest that it is reasonable to assume that obese patients (BMI > 30) with an ED and hospitalized severely malnourished patients with AN are at increased risk of developing a (silent) COVID-19 infection and suffer more complications. At the very least clinical vigilance should be raised when treating both patient groups.

After considering consequences of underdiagnosing COVID-19 on the individual level and the risk of nosocomial outbreaks, for both healthcare workers who provide direct care to inpatients and patients with AN considered for clinical admission, we advise preadmission RT-PCR testing even without clinical suspicions of infection and, in addition, when available and clinically indicated, antibody profile for patients. Since the sensitivity of RT-PCR, to date the ‘gold standard’ for the diagnosis of COVID-19, is moderate and asymptomatic patients may develop an infection during inpatient treatment, there is a need for daily clinical rescreening for signs and symptoms of infection and repeat testing on indication. Additionally, we recommend to educate patients to raise awareness of the need for screening and testing, and to avoid misinformation, fear and stigmatization. Undoubtedly, there are compelling reasons to effectively prevent the spread of COVID-19 in inpatient ED units. First, in congregate settings, COVID-19 can spread rapidly so prompt identification of COVID-19 cases and implementation of prevention measures are critical to ensure the protection of other patients and staff members. A report from New York (USA) showed an overall rate of COVID-19 infection of 15.6%, with an asymptomatic positive rate of 13.7% among patients in psychiatric inpatient settings from 1 March to 1 May 2020, which is higher than the 10.3% positivity in respiratory specimens from the broader US population at the same time [32]. Second, as mentioned above, reliance on the presence of symptoms to guide COVID-19 testing in AN can lead to under- or overdiagnosis. Also, several previously published reports of COVID-19 disease among psychiatric inpatients identified inconsistent and incomplete responses to survey questions because patients would often mention non COVID-19 like symptoms or attribute symptoms to existing comorbidities, leading to underdiagnosis in those who are COVID-19 positive, since both groups would not be tested on the basis of their survey responses. Additionally, it has been reported that only 26% of positive adolescent inpatients had typical symptoms [33]. Third, some recommended elements of contingency planning for psychiatric hospitals and residential settings, such as restricting dining to in-room meals instead of communal dining [32], are not feasible or therapeutic in AN inpatient settings.

In the outpatient setting patients should be screened regularly for symptoms and known exposure in between sessions. Indeed, several authors (e.g., [34]) recommend reducing the threshold for COVID-19 testing and, once vaccination is widely available, encouraging people with obesity to be vaccinated. Because the duration of virus shedding may be prolonged in obese outpatients having tested positive for COVID-19, the quarantine period for this population may need to be extended, with treatment comprised of telemedicine only.

There are currently more than 100 COVID-19 vaccine candidates under development. With astonishing speed, at least seven vaccines have achieved regulatory authorization or approval around the globe and there are at least 55 vaccine candidates in phase 1–3 clinical trials listed in the COVID-19 vaccine tracker of the Regulatory Affairs Professional Society (RAPS) site on 23 December 2020. All seven leading vaccines probably have a minimum 50% efficacy threshold, on the basis of clinical trial data so far. However, it is not clear how long the vaccines’ protective effects last, whether it can block people from transmitting the virus or whether one vaccine works better than another in certain groups of people.

Since poor vaccine-induced immune responses have been observed in the obese (e.g., hepatitis B, influenza H1N1) [35], there were concerns that COVID-19 vaccines would not work well for obese people. Fortunately, data from the the Pfizer/BioNTech and the Moderna COVID-19 vaccine trials showed around 95% efficacy in the participants at risk of severe COVID-19, including those with a BMI ≥ 30 [36, 37]. However, since conclusions about effectiveness are drawn from less than 200 participants with developed disease, more powered studies are needed to definitively confirm effects of vaccines across different demographics and conditions. Additionally, because vaccine supply will not be immediately available to immunize all who could benefit from vaccination, most countries have defined priority groups. For example, the provisional COVID-19 vaccine priority list, which outlines who will be given a vaccine first, published by Public Health England [38] is now divided into nine groups. People aged 16–65 with a BMI of 40 and above are in priority group six, meaning they will be prioritised for the vaccine ahead of healthy over-60s. Priority group six also includes adults aged 18–65 who suffer from other conditions considered to put them more ‘at risk’ for COVID-19. Although these include among others immunocompromised conditions and severe mental illness, AN is not yet treated as a priority group. Notably, currently there is no data yet on COVID-19 vaccine efficacy or safety in immunocompromised people. Nevertheless, The Centers for Disease Control and Prevention (CDC) and The European Medicines Agency (EMA) recommend these patients may still receive the vaccine and should be counselled they may have a lower immune response than the general population, and that the safety profile is not currently known. Comparable recommendations regarding the possible benefits and risks of COVID-19 vaccine in patients with AN appears to be lacking. A pilot study of immunogenicity of H1N1 vaccination among 10 adults with AN found antibody levels to be similar to normal weight counterparts, but the study lacked a control group and the persistence of immune response was not evaluated [39]. Hence, patients with AN and their physicians need to use these limited data to weigh the benefits and risks of COVID-19 vaccines, taking into account the patient’s specific risk for COVID-19. Importantly, as we cannot assume that data on one vaccine type in the obese and AN can be extrapolated to other vaccine types, it is therefore urgent that any COVID-19 vaccine studies include anthropomorphic measurements as a potential confounder for vaccine effectiveness. Companies that have developed COVID-19 vaccines may wish to include patients with AN in clinical trials and post authorization observational studies on vaccine use.

Together, research on viral detection, longitudinal follow-up of viral shedding, laboratory biomarkers (e.g., haematological markers, cytokines, immunoglobulins) and clinical manifestation and outcomes of COVID-19 in ED are necessary. To improve the understanding of the impact of COVID-19 infection on patients with ED, we strongly urge that all such patients be enrolled in patient (inter) national registries whenever possible. Data collection on patients with AN who received a COVID-19 vaccine will be needed to provide information of immunogenicity, safety and the persistence of immune response to guide future vaccines recommendations. As additional information from clinical trials and from data collected on vaccinated patients with AN outside clinical trials becomes available, it will be critical that ED professionals stay well informed about emerging data, safety and efficacy of vaccines so that they can help patients make sound decisions.

## Insights derived from findings on immunity in AN and obesity - future research directions

Striking similarities between the cytokine profile of COVID-19 infection and AN [12, 40], and recent findings on the role of T-cells in the immunity to COVID-19 [41] and its hypothesized role in viral resistance in AN [40, 42] may inspire future research.

While the question whether patients with AN are indeed less prone to viral infections than individuals with simple malnutrition remains to be empirically confirmed, the most recent reviews on the relationship between AN and immunity posit that this interplay is unique and more complex than it is in primary malnutrition (PM) [42]. Unlike what is seen in PM, patients with AN show intact or increased T-cell proliferation to various antigens, and an elevated CD4/CD8 ratio, while especially memory CD8 T-cell counts seem to be lower. Speculatively, this is related to a perceived lack of symptomatic common viral infections, as a marked reduction in memory CD8 T-cells could lead to a reduction in lymphocytes subserving recall responses. Although the majority of studies on cytokines in AN have yielded mixed and, at times, contradicting results, the most recent studies propose a unique immunological profile of AN. Of particular interest, pro-inflammatory cytokines (IL-1, IL-6, and TNF) appear to be elevated in AN when compared to PM, while the levels of the anti-inflammatory cytokine IL-10 seem to be significantly influenced by ED diagnosis and BMI (i.e., being higher in AN and normal-weight individuals and lower in binge-eating disorder and obesity) [43]. The overproduction of IL-10 and the down-regulation of pro-inflammatory cytokines could explain the absence of infection in AN.

Notably, several mechanisms that have been proposed to explain the relationship between obesity and COVID-19, such as low-grade chronic inflammation, impaired memory CD8 + T-cell responses, increased circulating levels of pro-inflammatory cytokines and hyperleptinaemia, overlap and mirror pre-existing models of the unique immunological and metabolic profile in AN in an opposite direction. Since AT is established as an immunological organ and emerging research identified significant negative genetic correlations between AN and obesity ([44], for review), we argue that the question why obesity is a risk factor for COVID–19 is inherently an evolutionary question that could be addressed by looking at the “opposite side of the same coin”, namely constitutional thinness, PM and possibly AN. Although constitutional thinness is a more evident “mirror image” of obesity than is AN and reconceptualization of AN as the opposite of obesity remains subject to vigorous debate, based on new genetic discoveries in AN it has been hypothesized that the conditions may represents “metabolic and microbiome bookends” [44]. Indeed, a genome-wide association study (GWAS) suggests that the genetic origins of AN are both metabolic and psychiatric. Additionally, the results indicated one GWAS-significant signal that has previously been linked to type-1 diabetes and autoimmune disorders, supporting an important new direction for research into some dimensions of speculative metabolic and immunological mirror images of AN and obesity ( [45], for review) that are shaped by evolutionary factors. Arguably, changes in food availability and infectious pathogens are probably among the strongest selective forces that act on the human genome. It has been hypothesized that infectious diseases favor investment in VAT because of its immune advantage in fighting infection [46], along with variable responses to specific infectious pathogens in terms of fat distribution and cytokine biology leading to the remarkable inter-individual and inter-population immune response and fat distribution heterogeneity [47]. Yet, some of the same mutations enabling humans to resist some infections also make us more prone to other infections and certain diseases, such as autoimmune disease, obesity and, possibly, AN. Although this assumption is not tested in AN, this hypothesis appears to be supported in other fields by evidence that having nonfunctional alleles of FUT2 protects against specific pathogens (e.g., Norovirus, Rotavirus, HIV) [48]. By contrast, the non-secretor phenotype has been also associated with increased susceptibility to other pathogens (e.g., Streptococcus pneumonia) and increased risk for certain autoimmune diseases, including type 1 diabetes and inflammatory bowel disease [48]. These examples serve to illustrate that the field of ED research could provide a unique opportunity to integrate the “metabolic and microbiome bookends” hypothesis of AN and obesity [44] and VAT-prioritization [46] and variable infectious disease selection hypotheses [47] into an overarching COVID-19 research framework aimed at investigating the susceptibility and resistance to virus infections in the whole spectrum of ED and obesity. A necessary first step will require prospective epidemiological, and clinical and laboratory studies on this subject. For example, examining whether variation of ACE2 expression, adipocyte viral tropism/load, viral shedding, serology markers, the cytokine and immune cells profile are different between the four major groups (obesity, constitutional thinness, simple malnutrition, AN /AN-R) could explain the differences in infection susceptibility.

## Conclusions

The COVID-19 pandemic brings much uncertainty and many challenges for clinicians treating patients with ED. However, sharing experiences is the best way to learn real-time lessons and adapt to this rapidly changing pandemic. There are important questions crucial to our understanding of susceptibility to, clinical manifestation, course and outcomes of COVID-19 in AN and obese ED patients that need to be answered in order to inform our testing, treatment and vaccination policy. Since our knowledge about COVID-19 is still evolving, the presented recommendations may need to change and our perspective and hypotheses should be seen as a focus for discussion to inspire further research.

As obesity appears to be an independent risk factor for COVID-19 and we are facing two pandemics: the ever-expanding obesity epidemic and the recent COVID-19 outbreak, we need to address both pandemics in the context of the genetic predisposition and the “obesogenic” niche applying modern evolutionary thinking. Combining research into AN and obesity with research into COVID-19 may provide a unique opportunity to study the susceptibility to COVID-19.

Given the low prevalence of AN and the widely dispersed but small patient base, efforts to engage the research and the patient communities to share their data are of critical importance. Creating a worldwide, shared database of viral and in particular COVID-19-related epidemiological, clinical (e.g., anthropomorphic measurement), laboratory and immunology data (e.g., serum cytokine changes, RT-PCR test results, antibody levels) in patients with ED and designing studies that explain the clinical observations may help to combat this and future pandemics. In addition, centralizing and integrating worldwide viral-related data with existing biobanks will be beneficial for future machine learning research to develop predictive diagnostic strategies and inform new hypotheses.

## Data Availability

Not applicable.

## Abbreviations

ACE2: Angiotensin-converting enzyme 2
AT: Adipose tissue
AN: Anorexia nervosa
AN-R: Recovered from anorexia nervosa
ARDS: Acute respiratory distress syndrome
BMI: Body mass index
CDC: Centers for disease control and prevention (CDC)
CRP: C-reactive protein
ED: Eating disorder(s)
EMA: European medicines agency
GWAS: genome-wide association study
PM: Primary malnutrition
RAPS: Regulatory affairs professional society
RT-PCR: Reverse-transcription polymerase chain reaction
VAT: Visceral adipose tissue
